# Completing asthma action plans by screen-sharing in video-consultations: practical insights from a feasibility assessment

**DOI:** 10.1038/s41533-020-00206-8

**Published:** 2020-10-21

**Authors:** Omer Hamour, Eve Smyth, Hilary Pinnock

**Affiliations:** 1 Blackpool Teaching Hospitals NHS Foundation Trust, Blackpool Victoria Hospital, Blackpool, UK; 2 Patient and Public Involvement Group, Asthma UK Centre for Applied Research, Usher Institute, The University of Edinburgh, Edinburgh, UK; 3 Asthma UK Centre for Applied Research, Usher Institute, The University of Edinburgh, Edinburgh, UK

**Keywords:** Patient education, Asthma

## Abstract

Supported self-management is a vital component of routine asthma care. Completion of an agreed personalised asthma action plan is integral to implementation of this care, and traditionally this requires a face-to-face consultation. We aimed to assess the practical feasibility and potential utility of using screen-sharing technologies to complete asthma action plans remotely. Assisted by people with diverse technological ability and using a range of devices, we tested the technological feasibility of completing action plans in remote consultations using two leading video-conference systems. We used a semi-structured topic guide to check functionality and lead feedback discussions. Themes were interpreted using the Model for ASsessment of Telemedicine applications (MAST). Discussions with ten participants (age 20–74 years) revealed that screen-sharing was practical on most devices. Joint editing of an action plan (as was possible with Zoom) was considered to encourage participation and improve communication. Attend Anywhere had less functionality than Zoom, but the NHS badging was reassuring. Most participants appreciated the screen-sharing and considered it enabled a meaningful discussion about their action plan. Online shared completion of action plans is feasible with only a few (potentially remediable) practical problems. These findings suggest this may be a fruitful approach for further study—made more urgent by the imperative to develop remote consultations in the face of a global pandemic.

## Introduction

An estimated 334 million people are living with asthma worldwide^1,2^, and integrating avoidance of triggers and the use of regular medication into their everyday lives to reduce the impact of their condition^3,4^. A vital self-management skill is the ability to recognise the signs and symptoms of deterioration and the action they should take^5^. Professionals support self-management by providing regular reviews, including patient education, reinforced by the shared completion of an agreed personalised asthma action plan^4^. There is overwhelming evidence that asthma self-management reduces the need for unscheduled care and improves quality of life^6^. Despite recommendations by national and global guidelines^3,4^, in many countries only about a third of people with asthma have an action plan^7–9^.

Although implementation in routine clinical practice is possible^10^, it is challenging not least because people with asthma do not always wish to give time to attending for a routine review when they are well^11,12^. Remote consultations are convenient and can improve access^13^ but may be criticised as not allowing shared completion of paper-based action plans. Increasing deployment of video-consulting^14,15^ offers the potential for a patient and a healthcare professional to complete an action plan together on a shared screen.

We aimed to assess the practical feasibility and potential utility of delivering action plans remotely using web conferencing and screen-sharing technologies.

## Results

### Initial scoping

The findings of our scoping of the screen-sharing aspects of Attend Anywhere and 11 widely used videoconferencing applications are summarised in Table 1. Originally, a third method of reviewing action plans in the form of Google Docs and a telephone call was considered; however, this method was deemed to be far inferior to videoconferencing and was therefore rejected.

**Table 1:** Summary of videoconferencing applications assessed in the scoping exercise.

Application	Free version?	Diverse operating system compatibility?	Diverse device compatibility?^a^	User-friendly?^b^	More in-app features relative to Attend Anywhere?^c^
Attend Anywhere	Yes	Yes	Yes	Yes	N/A
Zoom	Yes	Yes	Yes	Yes	Yes
TeamViewer	Yes	Yes	Yes	No	No
Mikogo	Yes	Yes	Yes	No	No
Skype	Yes	Yes	Yes	Yes	No
Zoho	Yes	Yes	Yes	Yes	No
Netviewer	No	No	No	No	No
GoToMeeting	Yes	Yes	Yes	No	No
Cisco WebEx	No	Yes	Yes	Yes	Yes
AnyMeeting	Yes	Yes	No	No	No
Livestorm	No	Yes	Yes	Yes	No

^a^Compatible with phones, tablets, and laptop/desktop.

^b^Adjudicated through trial use alongside a colleague. Parameters used include simplicity of navigation, aesthetic appeal, and responsiveness/loading times.

^c^For example: remote control, recording.

Two applications capable of screen-sharing in the context of a video-consultation were identified for further investigation: Attend Anywhere and Zoom.

- Attend Anywhere is a Google Chrome-based virtual clinic software promoted by NHS Scotland to increase healthcare service accessibility^16^.
- Zoom requires a one-time download and log-in. It has unique in-application features, including recording, in-app file transfer, and remote control, which offers the host/participant control of other’s mouse/keyboard^17^.

### Participants and arrangements for sessions

We recruited ten participants: five patient colleagues and five friends and family with a range of ages and using a range of devices (see Table 2 for details). The sessions, which lasted between 30 and 60 min, were conducted by O.H. who was at the time based in British Columbia, Canada, while the participants were situated in the UK, Canada, or Switzerland.

**Table 2:** Study participants and characteristics.

Participant ID	Age	Device used	AUKCAR PPI group?
P1	61	Macbook Air	No
P2	26	Microsoft Surface Pro	No
P3	20	Macbook Pro	No
P4	28	Macbook Pro	No
P5	55	iPhone 6 Plus	No
P6	40	iMac Desktop	Yes
P7	75	Dell Desktop	Yes
P8	60	iPad Mini 3	Yes
P9	50	iMac Desktop	Yes
P10	74	iMac Desktop	Yes

### Overview of findings

We identified two themes (summarised in Table 3):

- Technical aspects with sub-themes about functionality and connectivity, features of the videoconferencing softwares, and user-friendliness.
- Potential for delivery of care.

**Table 3:** Themes and sub-themes.

Themes	Sub-themes
Technical aspects	○ Feasibility and connectivity
	○ Features of the videoconferencing software
	○ User friendliness
Potential for routine care	○ Comparison to past experience
	○ Willingness to use
	○ Convenient but not such a good assessment

We describe our findings below with brief quotes from our field notes to highlight key points. A table with illustrative examples from our notes is in Supplementary Table 1.

### Technical aspects: functionality and connectivity

Eight out of ten participants were able to view the action plan through screen-sharing on both applications. The participant using the iPhone 6 plus was unable to see a picture on their screen using Attend Anywhere and was therefore unable to review the action plan, even after a second try. She was, however, able to review the plan on Zoom. Furthermore, the iPad Mini’s VoiceOver feature (used by a participant with visual problems), which typically reads the screen aloud, was unable to translate the contents of the shared screen using either system. The Asthma UK action plan is available both as a ‘PDF’ and a plain text version; neither worked with VoiceOver.

There were some connection problems with both systems. For example, Attend Anywhere ‘*froze*’, and required the user to ‘*go through the introduction screens again*’ to repair the call.

### Technical aspects: features of the videoconferencing software

Both softwares facilitated screen-sharing and provided in-meeting chat, which allowed users to send text messages to each other. File sharing and text chat were, however, unsuccessful in both applications when used through the iPhone 6 Plus and VoiceOver on the iPad Mini.

Zoom facilitated file sharing directly within the app, enabled recording of interactions, and allowed the participant to edit documents on the host’s screen using the remote control feature. The expediency of file sharing with Zoom was appreciated by some, but other participants, while recognising its usefulness, did not consider it provided overall increased benefit. One participant stated that they were satisfied with Attend Anywhere, which enabled them to ‘*talk to a nurse over the line*’ and ‘*forward the action plan by email after the consultation*’.

Most participants liked being able to record consultations, noting that it could help consolidate information delivered during the review, though others pointed out that the action plan was already a summary of what was discussed. Even with the action plan summary, ‘*additional points*’ may have been discussed that could be usefully reviewed in a recording.

Attend Anywhere does not allow the ‘patient’ to edit the document. This was the first software used and initially most participants did not mind this lack of functionality; it was enough to ‘*be able to see the document being completed*’. However, after trying the function in Zoom, participants identified several benefits of using the collaborative remote control feature. Some thought it would encourage them to ‘*take ownership*’ and participate in the revision of their action plan. Others felt that editing the document with the clinician improved communication, avoided misunderstandings, and ensured that the doctor or nurse ‘*picked up everything that was said*’. One participant thought that the ‘*process would go a lot quicker if they could help edit the document*’. This function did not work with VoiceOver.

### Technical aspects: user friendliness

Despite the technical limitations of both videoconference systems, most participants found them simple to navigate once opened. Some found it troublesome to access Attend Anywhere due to its sole compatibility with Google Chrome, which was not their default browser. On the other hand, other participants found Zoom’s one-time download requirement off-putting and appreciated Attend Anywhere’s simplicity, primarily because of its web-based nature that was perceived as easier for a ‘*non-technical user*’. There was a need for clarity about using the link—one participant was waiting for the researcher to call.

One participant explained that the content and layout of Attend Anywhere’s interface that was explicitly part of the NHS was an important factor for them. Features such as the NHS logo made it look ‘*more official*’, which helped them to ‘*trust that it was real*’. In contrast, others found that Zoom functioned better ‘*despite the same internet connection*’. VoiceOver proved to be more compatible with Zoom: Attend Anywhere ‘*described very little*’, whereas VoiceOver described the features available in the Zoom toolbar.

### Potential for routine care

Some participants voiced opinions about the potential for using screen-sharing in real-life consultations.

### Potential compared to past experience

Some participants who had received action plans in the past found very little difference between an online and a face-to-face consultation approach. The technology did not help the visually impaired patient overcome the problem of not being able to read a print action plan.

### Potential willingness to use

Nine of the ten participants said they would use video-conference software again. Some were very positive describing it as ‘*a really great idea*’ and commenting that being able to see the healthcare professional made it ‘*feel like a consultation*’ and that it was ‘*much better than doing it over the phone*’. Some considered that knowing the healthcare professional was an advantage suggesting that ‘*it would be fine if it was with my asthma nurse that I’ve seen for years*’.

### Potentially convenient but not such a good assessment

Most participants liked the screen-sharing approach because it increased accessibility (for example, if their mobility was compromised), efficiency, and offered convenience that ‘*outweighed the fact that they were not in the room with the healthcare professional*’. Many of the participants observed that assessment of their condition would be more difficult, so it might be less suitable during periods of ill health when there was concern that ‘*something else going on with my health*’ that might be overlooked in a video-consultation. In contrast, one participant observed that a video-consultation could be a ‘*life-saver*’ if they had an acute attack in a remote location and needed advice on ‘*what to do next*’.

## Discussion

Online screen-sharing was a practicable approach to joint completion of asthma action plans. Both softwares had some technical limitations: Zoom needed to download a piece of software, Attend Anywhere only worked with Chrome. Attend Anywhere had less functionality than Zoom, for example, not allowing the ‘patient’ to edit the action plan, but some found the NHS badging reassuring. Joint editing of an action plan (as was possible with Zoom) was considered to encourage participation and improve communication. Many participants observed that it was comparable to a live situation and considered that it offered a convenient, accessible, and efficient option.

The study has some strengths and limitations. A strength of this study was the practical trial of technology and the semi-structured feedback interviews, which allowed for a detailed evaluation of practical issues and other features concerning online review of action plans. Technology constantly develops, and we recognise that the systems we tried will have changed and additional functionality may now be available, though we are not aware that screen-sharing is being promoted as a feature in software designed for healthcare consultations suggesting that our findings may still be relevant.

The variety of ages, backgrounds, and technological abilities of study participants provided a multiplicity of perspectives. The sample size was small, but data saturation was achieved with respect to the key aim of exploring practical feasibility. Only one action plan was edited in the study, so the results may be different for other plans. A further limitation of the study is that the discussions were not recorded, which precluded reviewing and checking the accuracy of notes.

Finally, although our participants offered some opinions about the suitability of screen-sharing for joint completion of action plans in routine care, this was not a core aim of this study and participants were not recruited to represent the range of asthma patients—indeed half the participants did not have asthma. Their comments should be considered as offering ideas for exploration in a future study.

The Model for Assessment of Telemedicine applications provides a structure for assessing the efficacy and standard of care offered by telemedicine applications. The three elements are *preceding considerations*, *multidisciplinary assessment*, and *assessment of transferability*^18^, and these are considered in turn in our interpretation of our findings

Both applications used in this study facilitated the remote provision of action plans for the majority of participants, and most subjects found the approach to be comparable to a live situation. Furthermore, barriers to implementation were minimal as both applications are mature and used in real life; indeed Attend Anywhere is currently available for use in healthcare consultations in the UK and Australia. There is a long-standing precedent for the use of telephone consultations in asthma reviews^11^. Video-consultations are a relatively recent innovation enabling visual clues, which may improve rapport and communication^19^. Clinicians share the screen in face-to-face consultations as a strategy to involve patients in their healthcare^20^; our study shows that ‘screen-sharing’ in a video-consultation to complete an action plan is possible.

Action plans completed as part of a self-management discussion improves health outcomes of people with asthma, and participants noted that the online approach had the potential for time and cost-efficiency, convenience, and accessibility, which may facilitate implementation. From an organisational viewpoint, assessment of how an online approach would fit into routine practice would be needed before it was introduced. In the context of the UK, almost all action plans are provided by nurses, and almost all are delivered in face-to-face consultations^21^, with slow adoption of remote alternatives. This, however, has changed overnight with the coronavirus disease 2019 pandemic. The requirement for social distancing means that practices are adopting video-consultations to avoid face-to-face contact, and the widespread adoption of video-calling to maintain social contact during pandemic lockdowns means greater public familiarity with the technology^22^. Asthma UK’s advice that people with asthma should have an action plan will require remote strategies including potentially shared completion of an online plan.

Overall, participants were amenable to the screen-sharing approach, but broadening the compatibility of Attend Anywhere to include other web-browsers and expanding its feature profile may enhance user friendliness. Socio-cultural, ethical, and legal aspects were not evaluated in this study and are areas for future research.

NHS firewalls would be very unlikely to allow practices to use Zoom for consultations—especially if it allowed patients to control the mouse/keyboard on an NHS computer. Attend Anywhere is approved in the NHS, but the functionality does not permit editing or transferring the completed plan to the patient for saving locally—or printing, though our participants were not too concerned by this. The need to upgrade NHS technology infrastructure to overcome practical barriers to video-consulting (such as lack of bandwidth, interruptions to communication, no web-cams) has been described previously^20^.

In conclusion, joint completion of action plans using screen-sharing technology was feasible, with only a few (potentially remediable) practical problems. Attend Anywhere is approved and available within the NHS but has less functionality than Zoom; neither had considered the needs of people with visual problems. Most participants appreciated the screen-sharing and did not feel it diminished their discussion about the action plan. These findings suggest that this may be a fruitful approach for a further study—made more urgent by the imperative to develop remote consultations in the face of a global pandemic.

## Methods

### Ethical review

The study was conducted in April 2018 with University of Edinburgh ethical approval (Level 1) Project SSC5a1684913.

### Identifying and initial scoping of potential video-consultation applications

Eleven videoconferencing softwares with screen-sharing capabilities were assessed to identify features that would enable joint completion of action plans. Two applications were shortlisted, which seemed most promising for further investigation.

### Sampling and recruitment

Email invitations to participate were sent to colleagues, adult family members, and friends with a range of experience of using technology and using different digital platforms. Our aim was to evaluate the feasibility of using the technology, so there was no requirement to have asthma, though we included some lay colleagues from the Asthma UK Centre for Applied Research Patient and Public Involvement group who may have had experience of receiving paper action plans in traditional face-to-face consultations and would be able to compare with the traditional face-to-face approach. The first invitation was sent to gauge interest and included an information leaflet. Once any questions had been answered and written consent provided in an email response (participants were from several countries), arrangements were made for the video-conference session including instructions on accessing the two shortlisted applications.

### Data collection and analysis

The video-conference sessions, which lasted between 30 and 60 min, were conducted by O.H., a male medical student who was at the time based in British Columbia, Canada, while the participants were situated in the UK, Canada, or Switzerland. After explaining the study and confirming consent, O.H. shared his screen and attempted to complete the action plan recommended by UK guidelines^4^, noting practical issues with using the technology and following a topic guide with semi-structured questions (Supplementary Methods). O.H. and H.P. developed the original topic guide, which was evolved iteratively in the light of issues observed in early sessions.

In each session, participants clicked on the link provided in the email invitation to join the first meeting on Attend Anywhere. Once connected, an adult Asthma UK action plan was reviewed through screen-sharing, which initiated dialogue around the software, its usability, and its potential application in clinical practice. Participants were shown the Canadian and Australian action plans for comparison. Following an attempt at completing an action plan with Attend Anywhere, participants clicked on another link to join a meeting hosted on Zoom. The same action plan was completed with Zoom enabling comparison between the functionality with the two systems. Participants were reminded that the information completed on the action plan was for technical demonstration purposes only and that resultant plans should be destroyed at the end of the interview.

We did not record the sessions because the main aim was to assess the utility of the system, but notes were made of comments made during the sessions. The ‘quotes’ in this report are thus not verbatim but transcribed from notes made at the time of the video-conference sessions. These field notes were coded (by O.H.) and analysed thematically (O.H. in discussion with H.P.) using a framework approach to illustrate the observations made about the intricacies and implications of using screen-sharing technologies to complete action plans^23^.

### Reporting summary

Further information on research design is available in the Nature Research Reporting Summary linked to this article.

## Supplementary information

Supplementary Information

Reporting Summary

## Data Availability

Authors confirm that all relevant data are included in the paper and/or its Supplementary Information files. We do not have consent to share data; applications for additional information should be directed to the corresponding author.

## References

[1] Mukherjee M (2016). The epidemiology, healthcare and societal burden and costs of asthma in the UK and its member nations: analyses of standalone and linked national databases. BMC Med..

[2] Global Asthma Network. The Global Asthma Report 2014. http://globalasthmareport.org/2014/index.php (2014). Accessed June 2020.

[3] Global Initiative for Asthma. Global Strategy for Asthma Management and Prevention. https://ginasthma.org/ (2020). Accessed June 2020.

[4] Scottish Intercollegiate Guideline Network/British Thoracic Society. British guideline on the management of asthma. https://www.sign.ac.uk/sign-158-british-guideline-on-the-management-of-asthma (2018). Accessed June 2020.

[5] Pinnock H (2015). Supported self-management for asthma. Breathe.

[6] Pinnock H (2017). Systematic meta-review of supported self-management for asthma: a healthcare perspective. BMC Med..

[7] Wiener-Ogilvie S (2007). Do practices comply with key recommendations of the British Asthma Guideline? If not, why not?. Prim. Care Respir. J..

[8] Sulaiman N (2011). Written asthma action plans (WAAPS) in Melbourne general practices: a sequential mixed methods study. Prim. Care Respir. J..

[9] Centers for Disease Control and Prevention. National Asthma Control Program Asthma Call-back Survey. http://www.cdc.gov/asthma/ACBS.htm (2013). Accessed June 2020.

[10] Pinnock H (2015). Implementing supported self-management for asthma: a systematic review and suggested hierarchy of evidence of implementation studies. BMC Med..

[11] Pinnock H, Madden V, Snellgrove C, Sheikh A (2005). Telephone or surgery asthma reviews? Preferences of participants in a primary care randomised controlled trial. Prim. Care Respir. J..

[12] Pinnock H (2007). Accessibility, clinical effectiveness, and practice costs of providing a telephone option for routine asthma reviews: phase IV controlled implementation study. Br. J. Gen. Pract..

[13] Pinnock H (2003). Accessibility, acceptability, and effectiveness in primary care of routine telephone review of asthma: pragmatic, randomised controlled trial. BMJ.

[14] NHS England. Digital First primary care. https://www.england.nhs.uk/gp/digital-first-primary-care/ (2020). Accessed June 2020.

[15] Asthma UK. Digital asthma: re-imaging primary care. Available from https://www.asthma.org.uk/support-us/campaigns/publications/digital-asthma/ (2020). Accessed June 2020.

[16] Attend Anywhere. Make travel optional: empowering video call access to the health sector and beyond. https://www.attendanywhere.com/ (2020). Accessed June 2020.

[17] Zoom. Zoom meetings and chat. https://zoom.us/meetings (2020). Accessed June 2020.

[18] Kidholm K (2012). A model for assessment of telemedicine applications: MAST. Int. J. Technol. Assess. Health Care.

[19] Donaghy E (2019). Acceptability, benefits, and challenges of video consulting: a qualitative study in primary care. Br. J. Gen. Pract..

[20] Milne H (2014). Does sharing the electronic health record in the consultation enhance patent involvement? A mixed-methods study using multi-channel video recording and in-depth interviews in primary care. Health Expectations.

[21] Morrow S (2017). Exploring the perspectives of clinical professionals and support staff on implementing supported self-management for asthma in UK general practice: an IMP^2^ART qualitative study. npj Prim. Care Respir. Med..

[22] Reuters. Zoom pulls in more than 200 million daily video users during worldwide lockdowns. https://uk.reuters.com/article/us-health-coronavirus-zoom/zoom-pulls-in-more-than-200-million-daily-video-users-during-worldwide-lockdowns-idUKKBN21K1C7 (2020). Accessed June 2020.

[23] Braun V, Clarke V (2006). Using thematic analysis in psychology. Qual. Res. Psychol..

