# Supplementary material for: Completing asthma action plans by screen-sharing in video-consultations: practical insights from a feasibility assessment
Source: NPJ Prim Care Respir Med. 2020 Oct 21;30:48. doi: 10.1038/s41533-020-00206-8 (PMC7578638; doi:10.1038/s41533-020-00206-8)
Supplement: Supplementary file 1 — Supplementary Information [file 41533_2020_206_MOESM1_ESM.pdf]

Supplementary table 1. Exemplar field notes to illustrate the themes and sub-themes

Themes	Sub-themes	'Quotes' as recorded in field notes
Technical aspects	Feasibility and connectivity	Attend Anywhere is not working. I can hear you, but I can't see your picture. Zoom is working – I can hear you and see the action plan. (P5)<br>VoiceOver doesn't seem to work with Attend Anywhere; I can't tell what's on the screen. Zoom told me that I started screen sharing, but not what was on the screen. (P8)<br>Attend Anywhere disconnected once – it froze, and I couldn't see or hear anything. Refreshing the link worked, but I had to go through the introduction screen again to repair the call (P3)
	Features of the videoconferencing software	*General functionality*<br>Zoom's good. Better than email or Attend Anywhere because I could get the file right away. It's quicker. (P4)<br>Worked really well. Email works too, so there's not much benefit of sharing the file within the app. With Attend Anywhere I could talk to the asthma nurse over the line, and they could forward the action plan to me by email. (P10)<br>VoiceOver didn't mention anything about the file. It doesn't mention anything about chat. The file is difficult to get to. (P8)<br>*Recording feature*<br>Recording could be useful as you only hear things one time. I have had experiences before when I've gone to the help desk and asked for a copy of discussions we've had over email. (P10)<br>The whole point of the consultation is to fill in the action plan. If that's been done, there's no need to go over the consultation. If the consultation was for something else, recording could be useful. (P4)<br>*Ability for the 'patient 'to edit the action plan*<br>I have no problem with not being able to edit the document, as the fact that both of us can see it makes it alright not to be filled out myself. (P7)<br>The remote control feature on Zoom is interactive, and lets you take ownership of your condition. (P4)<br>It would be useful overall because sometimes the nurse or doctor wouldn't pick up everything that's said, and miscommunications can be confusing. It would be good if both had the power to edit. It would be useful for things like triggers. (P10)<br>Editing the document on Attend Anywhere should be an option. The process would go a lot quicker if I was able to edit the document with you. (P2)
	User Friendliness	I struggled to get onto Attend Anywhere in the first place because Google Chrome was not my default browser. (P10)

		The fact that I had to download Zoom was a bit of a pain. Attend Anywhere was easier to get into, and once it got running, it was OK. (P9)<br>Attend Anywhere was easier to set up and easier to use because it did not require me to download software. For a non-technical user, Attend Anywhere would be more beneficial. (P10)<br>Attend Anywhere had the NHS logo and looked more official, so I trusted that it's real. It feels more official than Zoom. From a trust point of view, I preferred Attend Anywhere. (P6)<br>Attend Anywhere seemed dodgy. It didn't function as well as Zoom, even though we were using the same internet connection. (P3)<br>Zoom was easy to use. I would put it number one because of file sharing. (P2)<br>Zoom was easy to use and get onto. I clicked the email link, and it went to Safari and I barely noticed it. There were no technical problems. The features bar tells a lot more than Attend Anywhere. I don't know if I would play around with all of the features, but VoiceOver told me more about them. Attend Anywhere described very little. I preferred Zoom, because it was easier to access, especially since I didn't have to go through Chrome. (P8)
Potential for Routine Care	Comparison to past experience	This online format is good, and quite like a live situation. There is no difference to doing it face-to-face, I feel it would be the same. (P7)
	Willingness to Use	I think this is a really great idea, I would love to do this. It feels like a consultation, and it's much better than doing it over the phone because I can see you. The convenience factor outweighs the fact that I'm not in the room with you. (P9)<br>If it was with my asthma nurse that I've seen for years, it would be fine. (P8)
	Convenient, but not such a good assessment	If I wasn't able to go to the doctor for some reason, for example if I lost my mobility, I would certainly use them. If I was unwell, I would rather see the GP/nurse in person. (P7)<br>You can see me, but you can't see how I am. There might be something else going on with my health that wouldn't be seen over videoconference. I wouldn't replace speaking to an asthma nurse in person for this technology. (P10)<br>It would be particularly useful in an emergency setting where an asthma attack comes on and you need to know what to do next, especially in a remote location. This would be a life-saver. (P10)

Supplementary Methods. Topic guide

Pre-Interview / Participant Identifiers

1) Age:
2) Occupation:
3) Device used (laptop/tablet/phone):
4) AUKCAR PPI Group?

Attend Anywhere

1) Is this working?
    a.
2) How does this compare to completing asthma action plans in person? What would you think if your asthma nurse did something like this? What would you think if this actually happened?
    a.
    b.
    c.
3) What do you think about not being able to edit the document yourself?
    a.
    b.
    c.
4) Which AAP do you prefer?
    a.
    b.
    c.
5) What do you think about Attend Anywhere in general? How easy was it to use?
    a.
    b.
    c.
6) Any technical or other difficulties/problems using this method?
    a.
    b.
    c.

7) Would you use this again?
    a.

Zoom

1) Is this working?
    a.
2) What do you think about the file sharing feature?
    a.
    b.
    c.
3) What do you think about the recording feature?
    a.
    b.
    c.
4) What do you think about the remote control feature?
    a.
    b.
    c.
5) What do you think about Zoom in general? How easy was it to use?
    a.
    b.
    c.
6) Any technical or other problems using this method?
    a.
    b.
    c.
7) Would you use this again?
    a.

Final comments

1) How was the experience for you?
    a.
    b.
    c.
2) Which method/software did you prefer, and why?
    a.
    b.
    c.
3) Have you been given an AAP in the past? *If so*, how did it differ from completing asthma action plans in person?
    a.
    b.
    c.

<u>Reminder: Destroy the action plans we have completed – they are for technology demonstration purpose only.</u>
